# Urine-Derived Stem Cells Express 571 Neuromuscular Disorders Causing Genes, Making Them a Potential *in vitro* Model for Rare Genetic Diseases

**DOI:** 10.3389/fphys.2021.716471

**Published:** 2021-10-20

**Authors:** Maria Sofia Falzarano, Rachele Rossi, Andrea Grilli, Mingyan Fang, Hana Osman, Patrizia Sabatelli, Manuela Antoniel, Zhiyuan Lu, Wenyan Li, Rita Selvatici, Cristina Al-Khalili, Francesca Gualandi, Silvio Bicciato, Silvia Torelli, Alessandra Ferlini

**Affiliations:** ^1^UOL (Unità Operativa Logistica) of Medical Genetics, University of Ferrara, Ferrara, Italy; ^2^The Dubowitz Neuromuscular Centre, UCL Great Ormond Street Institute of Child Health, London, United Kingdom; ^3^Department of Life Sciences, University of Modena and Reggio Emilia, Modena, Italy; ^4^Beijing Genomics Institute (BGI)-Shenzhen, Shenzhen, China; ^5^Department of Medical Microbiology, Faculty of Medical Laboratory Sciences, University of Khartoum, Khartoum, Sudan; ^6^CNR-Institute of Molecular Genetics “Luigi Luca Cavalli-Sforza”- Unit of Bologna, Bologna, Italy; ^7^Istituto di Ricovero e Cura a Carattere Scientifico (IRCCS) Istituto Ortopedico Rizzoli, Bologna, Italy; ^8^Department of Proteomics, KTH Royal Institute of Technology, Stockholm, Sweden; ^9^National Institute for Health Research, Great Ormond Street Institute of Child Health Biomedical Research Centre, University College London, London, United Kingdom

**Keywords:** neuromuscular disorders, neurodegenerative disorders, urine derived stem cells, RNA-seq, western blot (WB), immunofluorescence

## Abstract

**Background:** Neuromuscular disorders (NMDs) are a heterogeneous group of genetic diseases, caused by mutations in genes involved in spinal cord, peripheral nerve, neuromuscular junction, and muscle functions. To advance the knowledge of the pathological mechanisms underlying NMDs and to eventually identify new potential drugs paving the way for personalized medicine, limitations regarding the availability of neuromuscular disease-related biological samples, rarely accessible from patients, are a major challenge.

**Aim:** We characterized urinary stem cells (USCs) by in-depth transcriptome and protein profiling to evaluate whether this easily accessible source of patient-derived cells is suitable to study neuromuscular genetic diseases, focusing especially on those currently involved in clinical trials.

**Methods:** The global transcriptomics of either native or MyoD transformed USCs obtained from control individuals was performed by RNA-seq. The expression of 610 genes belonging to 16 groups of disorders (http://www.musclegenetable.fr/) whose mutations cause neuromuscular diseases, was investigated on the RNA-seq output. In addition, protein expression of 11 genes related to NMDs including *COL6A*, *EMD*, *LMNA*, *SMN*, *UBA1*, *DYNC1H1*, *SOD1*, *C9orf72*, *DYSF*, *DAG1*, and *HTT* was analyzed in native USCs by immunofluorescence and/or Western blot (WB).

**Results:** RNA-seq profile of control USCs shows that 571 out of 610 genes known to be involved in NMDs, are expressed in USCs. Interestingly, the expression levels of the majority of NMD genes remain unmodified following USCs MyoD transformation. Most genes involved in the pathogenesis of all 16 groups of NMDs are well represented except for channelopathies and malignant hyperthermia related genes. All tested proteins showed high expression values, suggesting consistency between transcription and protein representation in USCs.

**Conclusion:** Our data suggest that USCs are human cells, obtainable by non-invasive means, which might be used as a patient-specific cell model to study neuromuscular disease-causing genes and that they can be likely adopted for a variety of *in vitro* functional studies such as mutation characterization, pathway identification, and drug screening.

## Introduction

Neuromuscular diseases (NMDs) are a broadly defined collection of rare inherited degenerative diseases affecting spinal cord, peripheral nerve, neuromuscular junction and muscle. The genetic diagnosis for NMDs has rapidly improved, due to the recent development of technologies such as next-generation sequencing (NGS) that accelerated the discovery of novel NMD phenotypes and genotypes associated with new classes of mutations (Tian et al., 2015). To date, more than 600 genes have been reported to cause NMDs^1^ and are a potential target for personalized medicine. Indeed, the consequent identification of new pathogenic targets and the related biological processes led to the discovery of novel therapeutic strategies for disorders that have been considered, for a long time, untreatable. Among 200 molecules in preclinical and clinical stages for NMDs, the majority target Duchenne muscular dystrophy (DMD) and amyotrophic lateral sclerosis (ALS) (Cowling and Thielemans, 2019). Gene therapy approaches are being tested in clinical trials for patients with mutations in *DMD*, *SOD1* (Superoxide dismutase 1, soluble) or *C9orf72* (Chromosome 9 open reading frame 72) genes^2^ (Cappella et al., 2019; Fortunato et al., 2021). Multiple FDA-approved *SMN* (Survival of motor neuron)-targeting treatments have been also developed for spinal muscular atrophy (SMA) (Dowling et al., 2018; Al-Zaidy and Mendell, 2019).

Despite these recent developments, potential drugs are not available for most NMDs. The progress in this field is strictly dependent on and supported by preclinical studies, and the availability of cellular and animal models to accurately recapitulate the disease phenotype is a crucial and limiting aspect for discovering and developing drugs for NMDs.

Different cellular models have been characterized to improve molecular diagnosis and functional studies in these disorders. Currently procedures used to generate patient-specific cells such as myogenic cells, fibroblasts, and induced pluripotent stem cells (iPSCs) are mainly based on invasive methods (i.e., skin and muscle biopsies) (Speciale et al., 2020). Increased attention has been focused on stem cells derived from urine specimens (USCs) that now are used in a broad field of applications and, in some cases, are replacing the traditional cell sources obtained with invasive and time-consuming methods (Falzarano and Ferlini, 2019). USCs are a subpopulation of urinary system derived cells with stem cell properties including high proliferative capacity, multipotency, and immunomodulatory ability.

Native USCs, direct reprogrammed USCs, and especially USCs-induced pluripotent stem cells were used for studying several genetic diseases. Interestingly, native USCs obtained from patients affected by Fabry disease, inherited epidermolysis bullosa, or spinal muscular atrophy exhibited typical disease markers (Schosserer et al., 2015; Zhang et al., 2017; Slaats et al., 2018).

Despite these previous studies showed great interest in the USCs model, few data are available in the NMD field regarding their detailed transcription and protein characterization. It has been shown that USCs represent an ideal source for studying DMD and limb-girdle muscular dystrophy (LGMD) type 2 diseases (Falzarano et al., 2016; Kim et al., 2016), suggesting that they might have great potential for modeling other neuromuscular diseases.

Here, we have specifically interrogated the RNA-seq output for the expression of 610 genes in which mutations cause NMDs. We showed that the vast majority of NMD genes (93%) are transcribed in both native and MyoD transformed USCs, and that different groups of NMDs are overall represented with different gene expression rates. Following the myogenic transformation of USCs, the expression of only a few genes is modified.

We then validated RNA-seq data by immunofluorescence and/or western blotting (WB) for some selected genes causing muscular dystrophies such as *COL6A* (Collagen Alpha 1, 2, and 3 type), *EMD (*Emerin), *LMNA (*Lamin A/C), *DYSF* (Dysferlin), and *DAG1 (*Dystroglycan 1), and another set of genes causing motor neuron diseases such as *SMN*, *UBA1* (Ubiquitin-activating enzyme 1), *DYNC1H1* (Dynein, cytoplasmic 1, heavy chain 1), *SOD1*, and *C9orf72*.

By immunofluorescence, we found that COL6A protein is deposited in the extracellular matrix (ECM), and by WB analysis we confirmed that it is secreted in the cell medium. WB also demonstrated the translation of all the other tested genes, including those with lower transcriptional expression levels such as *SMN* and *C9orf72*.

Lastly, we extended the protein analysis to the *HTT* (Huntingtin) gene causing Huntington’s disease (HD), as an example of a neurodegenerative disorder for which modeling pathological processes in patient-specific cells is not without challenges. Interestingly, we found that USCs also synthesize the HTT protein.

The results of our work demonstrate that USCs could be considered an appropriate *in vitro* model to test COL6-myopathies (Bethlem myopathy and Ullrich congenital muscular dystrophy), Emery-Dreifuss muscular dystrophies, SMA, ALS, Charcot-Marie-Tooth disease, Muscular dystrophy-dystroglycanopathy, and HD, and we suggest that their use could be expanded to more than 90% of known NMDs if further studies on patients’ USCs will confirm our results.

The availability of a new *in vitro* model, easily obtainable from NMDs patients and able to recapitulate the hallmark features of the diseases, should accelerate the discovery of new candidate therapeutic molecules in pre-clinical stages and translate them into clinical studies.

## Materials and Methods

### Isolation of Human Urinary Stem Cells

Urine samples were obtained from three healthy controls (age of subjects: 42, 49, and 29) and USCs were derived and cultured as previously described (Falzarano et al., 2016).

Briefly, urine samples were obtained from each subject and processed within 4 h from the collection.

The urine specimens were centrifuged at 400 × g for 10 min at room temperature, and washed with phosphate-buffered saline (PBS; Thermo Fisher Scientific, Waltham, MA) supplemented with an antibiotic/antimycotic solution (Sigma-Aldrich, St. Louis, MO). After discarding the supernatant, 1 ml of primary medium was added, and each sample was plated into a coated plate with 0.1% gelatin (Millipore, Billerica, MA). The primary medium was removed 96 h after plating, and 1 ml of proliferation medium was added to 1 ml of primary culture medium in each well.

Primary medium: Dulbecco’s modified Eagle’s medium (DMEM)/high glucose (EuroClone, Pero, Italy) and Gibco Ham’s F12 nutrient mix (1:1; Thermo Fisher Scientific), supplemented with 10% (v/v) fetal bovine serum (FBS), antibiotic/antimycotic solution (Sigma-Aldrich), and an REGM (renal epithelial cell growth medium) SingleQuot kit (Lonza, Basel, Switzerland).

Proliferation medium: REGM BulletKit + RE cell basal medium (Lonza) and mesenchymal proliferation medium [DMEM/high glucose, 10% (v/v) FBS, 1% (v/v) Gibco GlutaMAX, 1% (v/v) non-essential amino acids (Gibco NEAA), 1% antibiotic/antimycotic solution, basic fibroblast growth factor (bFGF, 5 ng/ml; ProSpec, Rehovot, Israel), platelet-derived growth factor (PDGF-AB, 5 ng/ml; ProSpec), epidermal growth factor (EGF, 5 ng/ml; Lonza)] mixed at a 1:1 ratio.

The three native control USCs were used for both RNA-seq and protein analysis.

Myogenesis of native USCs was induced by infection with an adenovirus serotype 5 (Ad5)-derived, EA1-deleted adenoviral vector carrying the MyoD gene, as previously described (Spitali et al., 2009).

### Transcriptional Profiling of Urinary Stem Cells by RNA-Seq Analysis

We analyzed the gene expression levels of native USCs (WT-n) and MyoD-USCs (WT-m) in RNA derived from a pool of the three healthy controls.

We analyzed a pool of three different donors in order to reduce the biological variations among samples and to be sure that the transcriptional profile was not affected by the variability among donors. Total RNA was isolated using the RNeasy-kit (Qiagen, Chatsworth, CA) according to the manufacturer’s instructions. Libraries were prepared using TruSeq Kit (Illumina) according to the manufacturer’s instructions. The quality and quantity of the RNA library was assessed using the Agilent 2100 Bioanalyzer and the ABI StepOnePlus Real-Time-PCR System. RNA-seq was carried out with the Illumina HiSeq4000 at the Beijing Genomics Institute (BGI, Beijing). Read quality was verified using fastQC (v. 0.11.3).^3^ Raw reads were trimmed for adapters and for length at 100 bp with Trimmomatic, resulting in about 22 M (range 14.8–31.7 M) trimmed reads per sample. Reads were subsequently aligned to the human reference genome (GRCh38) using STAR (v. 2.5.3a; Dobin et al., 2013). Raw gene counts were obtained in R-3.4.4 using the *featureCounts* function of the *Rsubread* R package (v. 1.30.3; Liao et al., 2014) and the Gencode gene annotation. Raw counts were normalized to FPKM (Fragment Per Kilobase Million) using the edgeR package (Robinson et al., 2010).

Genes were categorized into four groups based on the quantiles of the FPKM distributions. Specifically, we defined high those genes with FPKM values higher than the 90th percentile of the distribution of FPKM values; middle those genes with FPKM values comprised between the 80th and the 90th percentile; low those genes with FPKM values comprised between the 50th and the 80th percentile, and not expressed the remaining genes with FPKM lower than the median FPKM values.

### Immunofluorescence

Control USCs were grown until confluent onto coverslips, and the medium was supplemented with 0.25 mM ascorbic acid 24 h before cell harvesting. For long-term cultures, confluent cells were grown for 6 days in the presence of 0.25 mM ascorbic acid, the medium being changed every 2 days. The immunofluorescence analysis of collagen VI was performed using an antibody against the collagen VI α3 chain, globular domain (Squarzoni et al., 2006) (clone 3C4, Millipore) and two polyclonal anti-collagen VI antibodies (70R-CR009X, Fitzgerald; ab6588, Abcam) followed by incubation with anti-rabbit and anti-mouse FITC-conjugated antibodies (DAKO). Samples were observed with a Nikon epifluorescence microscope.

### Western Blot Analysis

WB analysis of COL6A, USC culture medium (treated for 24 h with 0.25 mM ascorbic acid in the absence of FBS) was resolved by standard SDS–PAGE, electro-blotted onto a nitrocellulose membrane and incubated with the 70R-CR009X and a specific anti-α1 chain antibody (sc-20649, Santa Cruz), followed by incubation with anti-mouse or anti-rabbit horseradish peroxidase (HRP)-conjugated secondary antibodies. Chemiluminescent detection was carried out with the ECL detection reagent Kit (GE Healthcare Amersham) according to the supplier’s instructions.

All the other analyses were performed using the following method. USCs cells from the three healthy controls (ctrl1, ctrl2, ctrl3) were collected in lysis buffer (urea 4 M, Tris 125 mM pH6.8, SDS 4%) containing protease and phosphatase inhibitors (Roche, Merck United Kingdom). Protein quantification was performed using the Pierce BCA kit (Thermo Fisher Scientific, United States). Proteins were separated using NuPAGE 3–8% tris-acetate or NuPAGE 4–12% Bis-Tris gradient gels (Thermo Fisher Scientific) and transferred onto nitrocellulose membrane (Amersham Protran, GE Healthcare, United Kingdom). Membranes were blocked for 1 h at room temperature (RT) in 10% non-fat milk TBS-T (TRIS buffered saline + 0.1% Tween20) or in 3% BSA in 100 mM NaCl, 20 mM Tris pH 7.4. They were then incubated overnight at 4°C with primary antibodies (detailed in Table 1).

**TABLE 1:** List of antibodies targeting the NMD genes studied by Immunofluorescence (IF) and Western blot (WB).

Gene	Human Disease	OMIM	Primary antibody	Specie	Catalogue#	Dilution
***COL6A1***	− Bethlem myopathy − Ullrich congenital muscular dystrophy	# 158810 # 254090	(a) COL6A1 (b) Col6A1-A3 (c) Collagen VI (d) Collagen VI	(a) Rabbit polyclonal (b) Rabbit polyclonal (c) Rabbit polyclonal (d) Mouse monoclonal	(a) sc-20649 (b) 70R-CR009X (c) ab6588 (d) MAB1944	(a) 1:1000 (WB) (b) 1:1000 (WB) 1:100 (IF) (c) 1:100 (IF) (d) 1:50
***C9orf72***	Amyotrophic lateral sclerosis and/or frontotemporal dementia	# 105550	C90rf72	Mouse IgG2a	GTX634482	1:500
***SMN1***	Spinal muscular atrophy	# 253300 # 253550 # 253400 # 271150	SMN	Mouse IgG1	610647	1:500
***DYNC1H1***	− Charcot-Marie-Tooth disease, axonal, type 20 − Spinal muscular atrophy, lower extremity, autosomal dominant	# 614228 # 158600	DYNC1H1	Rabbit polyclonal	ABT266	1:5000
***UBA1***	Spinal muscular atrophy, distal, Xlinked, related to UBA1	# 301830	UBA1	Rabbit polyclonal	Orb411973	1:1000
***SOD1***	Amyotrophic lateral sclerosis	# 105400	SOD1	Rabbit polyclonal	10269-1-AP	1:1000
***DAG1***	− Muscular dystrophy-dystroglycanopathy (limb-girdle) − Congenital muscular dystrophy with hypoglycosylation of dystroglycan type A9	# 613818 # 616538	αDG IIH6 βDG	Mouse IgM Mouse IgG2a	05-593 B-DG-CE	1:2000 1:100
***DYSF***	− Miyoshi myopathy − Muscular; dystrophy, limb-girdle, type 2B	# 254130 # 253601	Dysferlin	Mouse IgG1	NCL-Hamlet	1:2000
***HTT***	Huntington Disease	# 143100	Huntingtin	Rabbit polyclonal	EPR5526	1:10000
***EMD***	Emery-dreifuss muscular dystrophy 1	# 310300	Emerin	Mouse IgG1	ab204987	1:1000
***LMNA***	− Charcot-Marie-Tooth disease, axonal, type 2B1 − Hutchinson-Gilford progeria syndrome − Mandibuloacral dysplasia with type a lipodystrophy − Restrictive dermopathy; lipodystrophy, familial partial, type 2 − Cardiomyopathy, dilated, 1A − Emery-Dreifuss muscular dystrophy , autosomal dominant.	# 605588 # 176670 # 248370 # 275210 # 151660 # 115200 # 181350	Lamin A/C	Rabbit monoclonal	ab108595	1:10000

*# phenotype description, molecular basis known.*

The following day, after 3 × 10 min washes in buffer at room temperature, membranes were incubated with the appropriate secondary antibodies: donkey anti-rabbit or donkey anti-mouse IRDye^®^ (1:10,000, Li-Cor, United States) for 1 h at RT. After 3 × 10 min washes in buffer at RT, membranes were imaged using the Odyssey infrared imaging system (Li-Cor, United States). Since the main aim was to investigate if there was or not protein expression, only one or two gels were run for each antibody with one replicate for each individual.

## Results

### Expression Profiling of Neuromuscular Genes

In total, we identified the transcripts of 20,716 genes in the two conditions, WT-n (native) and WT-m (MyoD) USCs. These 20,716 genes were categorized into four groups based on the distributions of the FPKM values. Specifically, we identified 2,071 genes with high expression (i.e., with FPKMs higher than 31.19 and 27.16 for WT-n and WT-m, respectively), 2,071 genes with middle expression (i.e., with FPKM values in the range 31.19–15.54 and 27.16–13.26 for WT-n and WT-m, respectively), 6,215 genes with middle expression (i.e., with FPKM values in the range 15.54–2.73 and 13.26–2.76 for WT-n and WT-m, respectively), and 10,358 not expressed genes (i.e., with FPKM values lower than 2.73 and 2.76 for WT-n and WT-m, respectively). All genes with the respective FPKM values and expression categories are listed in Supplementary Table 1.

**FIGURE 1:**
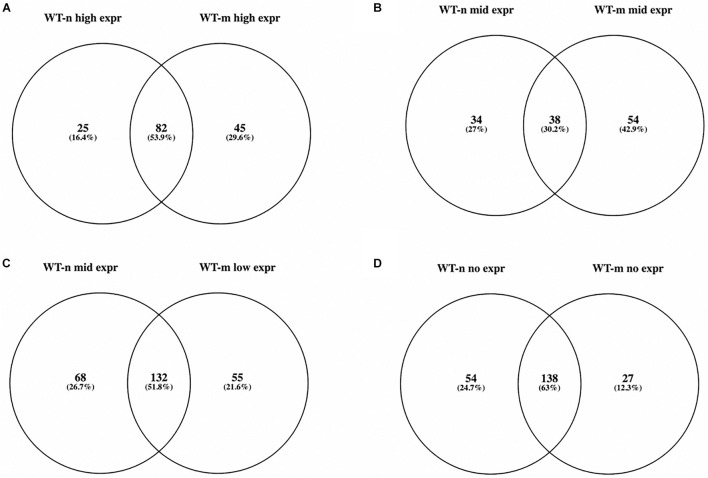
Venn diagram showing overlap of expressed NMD genes belonging to the four groups of expression in WT-n and WT-m USCs (**A:** high; **B:** mid; **C:** low; **D:** no expr). More than 50% of NMD genes at high and low expression were shared between WT-n and WT-m **(A,C)**, while only the 30% of NMD genes at middle expression were in common in both samples **(B)**.

We then focused on 610 genes known to cause neuromuscular diseases and belonging to 16 different groups of disorders (see text footnote 1, accessed 16 March 2021; Supplementary Table 2). We found that 571 of these NMD genes were present in the WT USCs gene expression data matrix (Supplementary Table 3) and that 379 and 406 of them were at high, middle, and low expression in WT-n and WT-m USCs, respectively. In particular, WT-n and WT-m had in common more than 50% of NMD genes at high and low expression, while only the 30% of NMD genes at middle expression were shared by both samples (Figures 1A–C). Interestingly, WT-m modified the expression levels of 154 genes (about 25% of total NMD genes) that resulted over-represented following the myogenic differentiation. Among the NMD genes that resulted not expressed in WT USCs, 138 were not expressed in both WT-n and WT-m, while 54 and 27 genes were specifically not expressed in WT-n and WT-m, respectively (Figure 1D).

The expression of few NMD genes (*n* = 43) either not expressed or with a weak expression in native USCs was restored following the myogenic differentiation such as *DES* (Desmin), *SGCD* (Delta-sarcoglycan), *SGCA* (Alpha sarcoglycan), *DTNA* (Dystrobrevin, alpha), *MYL1* (Myosin, light polypeptide 1, alkali, skeletal fast), *STAC3* (SH3 and cysteine rich domain 3) (Supplementary Table 3). Conversely, some genes that normally are not expressed in myogenic cells were, as expected, downregulated by myogenic differentiation as *COL6A* (Braghetta et al., 2008), *PLEKHG5* (Pleckstrin homology and RhoGEF domain containing G5) (Kim et al., 2013), and *SPTBN2* (Spectrin beta, non-erythrocytic 2) (Perkins et al., 2016) genes (Supplementary Table 3).

Transcripts related to the vast majority of disease groups were well represented in both native and myogenic USCs except for those associated with ion channel muscle diseases and malignant hyperthermia that were not expressed in either cell type as shown in Supplementary Table 4. Specifically, both WT-n and WT-m USCs did not express genes encoding sodium channel (*SCN4A*), chloride channel (*CLCN1*), calcium channel (*CACNA1S, CACNA1A*), potassium channel (*KCNJ18, KCNA, KCNE3*) and Na^+^/K^+^-ATPases (*ATP1A2*).

Among muscular dystrophies, LGMD and Emery-Dreifuss (EDMD) dystrophies are examples of diseases whose majority of the genes was expressed in native and/or MyoD USCs like *DES* and *TTN* (Titin) causing LGMD, *SYNE1* (Spectrin repeat-containing nuclear envelope protein) involved in the EDMD, *DYSF* (Dysferlin) associated with LGMD2B and Miyoshi myopathy (Supplementary Table 4).

The genes related to SMA and ALS, included in the Motor Neuron Diseases group, are all expressed in WT USCs. In particular, *SMN1*, that causes the most common form of SMA (types 1–4), is expressed in both native and myogenic-induced USCs. *UBA1* (Ubiquitin-activating enzyme 1) (Baumbach-Reardon et al., 2008), *DYNC1H1* (Dynein cytoplasmic 1 heavy chain 1) (Juntas Morales et al., 2017), *ASAH1* (N-acylsphingosine amidohydrolase (acid ceramidase) 1) (Topaloglu and Melki, 2016), and *GARS* (Glycyl-tRNA synthetase) (Antonellis et al., 2003) genes related to other SMA phenotypes showed high expression levels in both native and myogenic USCs.

Regarding ALS, most genes involved in the different phenotypes are very well expressed such as *SOD1*, *VCP* (Valosin-containing protein), *SQSTM1* (Sequestosome 1), *HNRNPA1* (Heterogeneous nuclear ribonucleoprotein A1), and *PFN1* (Profilin 1).

### Protein Analysis

**FIGURE 2:**
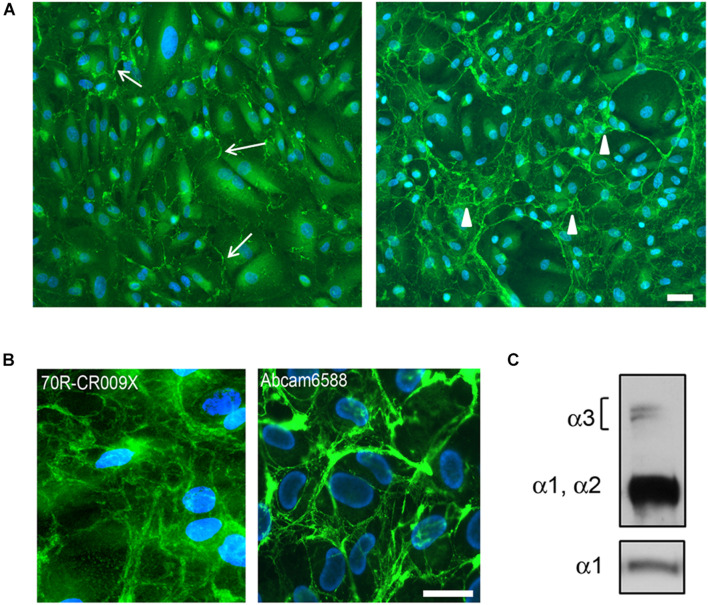
**(A)** Immunofluorescence analysis of COL6A protein in control USC cultures after L-ascorbate treatment for 24 h (left panel) and 6 days (right panel), with 70R-CR009X antibody showing filamentous (arrows) and web-like arrangement (arrowheads) of protein deposited in the extracellular matrix. Nuclei were stained with DAPI. Scale bar, 40 μm. **(B)** High magnification of COL6A protein obtained with two different antibodies, as indicated, showing the organization in web-like structures. Nuclei were stained with DAPI. Scale bar, 20 μm. **(C)** WB analysis of USC culture medium after treatment with L-ascorbate. COL6A protein was detected by immunoblotting with antibodies recognizing either all COL6A chains (Fitzgerald 70XR95) or the α1chain (Santa Cruz sc-20649).

To assess the dynamics of the extracellular assembly of collagen VI protein, native USC cultures were grown to confluence and treated for 24 h (short-term) or 6 days (long-term) with ascorbate. Using a polyclonal antibody (70R-CR009X), immunofluorescence microscopy revealed that collagen VI was already present in the matrix of USCs after short-term treatment, however, an extensive filamentous network was only apparent in long-term cultures (Figures 2A,B). A similar pattern was obtained with the ab6588 polyclonal antibody (Figure 2B), while the anti-α3 chain antibody (1944 Millipore) and an anti-N2-N7 α3(VI) antibody (Sabatelli et al., 2011) (data not shown) did not recognize COL6A protein in USCs. On the other hand, evidence of COL6A heterotrimer secretion was confirmed by WB analysis of the cell medium, which demonstrated the presence of clear bands at the predicted molecular weights of the α3, α1, and α2 chains (Figure 2C).

**FIGURE 3:**
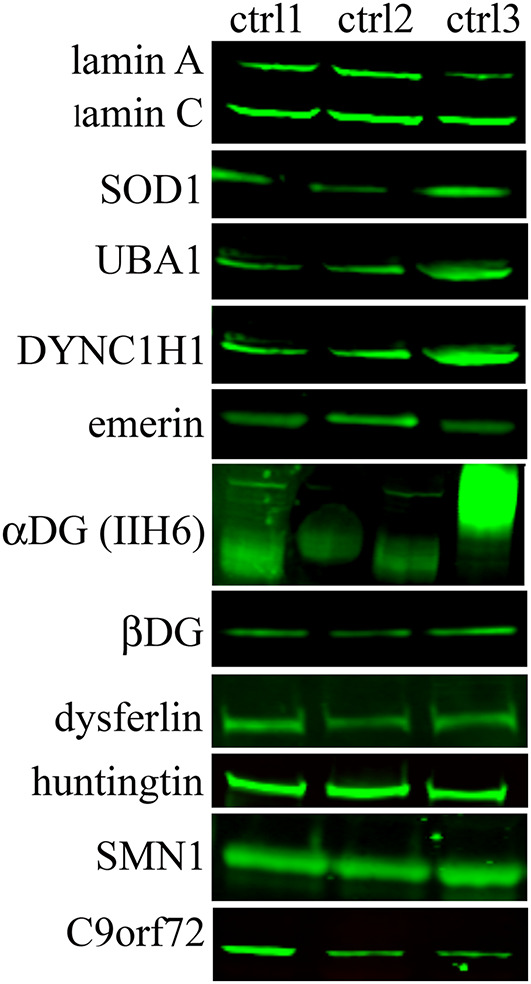
Protein expression in native USCs from three control individuals (ctrl1, ctrl2, and ctrl3) on WB. A representative image of protein results are shown from the top to the bottom based on their transcript expression (from the highest to the lowest FPKM value, Table 2). For DAG1, two antibodies (IIH6 and βDG) were used since this gene encodes for two proteins, α- and β-dystroglycan. IIH6 antibody binds to a specific glycan epitope on α-DG. For comparison, in the 4th lane the glycosylation in human skeletal muscle is shown.

The WB analysis on native USCs (Figure 3) for all the other selected genes (Table 1) showed bands, at the molecular weight predicted, for all the antibodies tested. We detected the proteins for all the four categories of expression (Table 2), including the genes belonging to the group with the lower FPKM values. Since the *DAG1* genes encodes for dystroglycan, a protein that is then cleaved into two proteins (α- and β- dystroglycan) (Brancaccio, 2019), we tested the expression of both proteins. To assess α-DG, an antibody (IIH6) that binds to a specific glycan epitope on α-DG was used and for comparison a skeletal muscle lysate was also loaded (4th bands, Figure 3).

**TABLE 2:** List of genes included in the protein analysis.

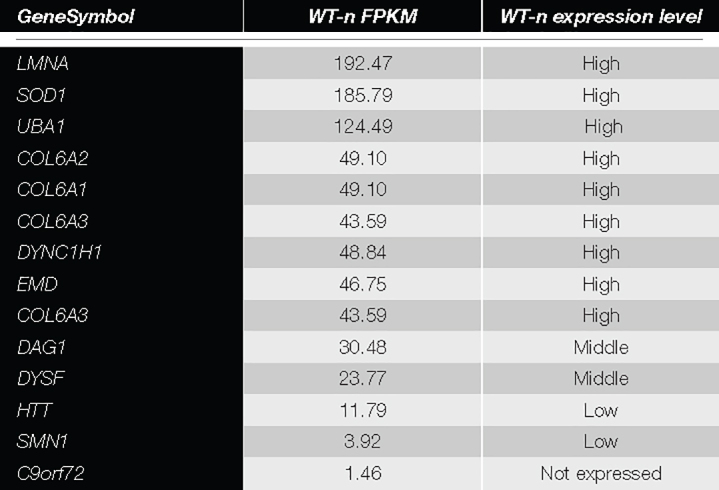

*Genes were categorized into four groups based on the quantiles of the FPKM distribution, i.e., high (dark gray), medium (gray) and low (light gray) expression and not expressed (white).*

## Conclusion

Understanding the pathophysiologic mechanisms underlying rare genetic diseases is essential for the development of new therapeutic approaches. Therefore, a realistic disease model able to faithfully recapitulate the pathology is crucial. Unfortunately, the lack of *in vitro* human disease or even patient-specific models often forces the use of animal models, mostly mice, which present challenges due to genetic and physiological differences between humans and animals, resulting in poor efficacy and safety in clinical trials (Sato et al., 2019). In addition, the EC Directorate has strongly encouraged the use of non-animal models and it has recently published the first review of strict legislation designed to protect research animals.^4^

Finding an easily obtainable source of patient-specific cells, especially for diseases involving inaccessible tissues such as skeletal and cardiac muscles and the central nervous system, would overcome these limitations and improve the progress in the field of a personalized therapy.

In a previous study, we demonstrated the applicability of USCs as a non-invasive and unlimited cell source modeling DMD (Falzarano et al., 2016), but only a few studies have evaluated the usefulness of USCs in modeling other NMDs (Falzarano et al., 2016; Sato et al., 2019).

The aim of our study was to determine whether USCs might represent a cellular model for other NMDs.

We characterized the gene expression profile of 610 genes causing NMDs in native and myogenic transformed WT USCs. Interestingly, we found that 571 transcripts were expressed in WT USCs, suggesting that USCs have the potential to model a variety of NMDs, at least at the transcriptional level. Indeed, the vast majority of NMD groups were well represented, and a few genes only showed a weak or absent expression in both WT-n and WT-m. Examples of these are ion channel muscle disease genes and malignant hyperthermia-related genes. Both native and MyoD cells did not express sodium, chloride, calcium, and potassium channel mRNAs. Therefore, USCs are not an appropriate tool to study pathogenic pathways or mutation effects of these genes. Nevertheless, it has been reported that USCs from a patient with a mild form of type 2 long QT syndrome can be reprogrammed in iPSCs that differentiate in functional cardiomyocytes recapitulating cardiac arrhythmia phenotypes caused by mutation in *KCNH2* gene (Jouni et al., 2015). Accordingly, USCs can be considered an attractive source to generate iPSCs for studying human ion channel diseases and other diseases as well.

When grouping genes based on their expression levels, we found that the myogenic transformation of USCs modified the expression levels of a limited set of high, middle, and low expression genes, and only a few genes that were not expressed in native USCs reverted to a positive expression. These genes, such as *DES*, *SGCD* and *SGCA*, *DTNA*, *MYL1*, *STAC3*, are, unsurprisingly, related to muscle function. This finding suggests that direct reprogramming of USCs into a myogenic cell line is required for some transcripts closely related to muscle function to be produced. Conversely, native USCs represent an ideal cell model for genes mainly expressed in the extracellular matrix, that are poorly represented in myogenic cells, such as *COL6A*, pathogenic variations in which cause Bethlem and Ulrich diseases (Tagliavini et al., 2014). This speculation is corroborated by our immunofluorescence and WB data, suggesting that USCs are a potential *in vitro* tool to study *COL6A*, as they are able to produce and secrete the fibrillary collagen VI protein.

In addition, differently from skin fibroblasts, antibodies against the α3 chain (N2–N7, and globular domain) did not recognize collagen VI in USCs. On the other hand, the α3 chain was secreted, as confirmed by WB analysis under denaturating conditions, which showed a doublet at 250 kD. These data point to additional post-translational modifications or conformational changes of α3(VI) in USC cultures. The expression of collagen VI chains is highly regulated at different levels and the α3(VI) N-terminus has several potential glycosylation sites which could generate additional molecular heterogeneity by attachment of branched oligosaccharides (Lampe and Bushby, 2005).

A similar finding was obtained using the antibody against alpha-dystroglycan (IIH6) directed against a carbohydrate epitope (Ervasti et al., 1997) that gives a band at a lower molecular weight in the USCs compared to muscle tissue, showing that a less glycosylated form of α-dystroglycan is expressed in the USCs. These modifications may be tissue/cell-type specific and, as for the different transcript levels, related to the differentiation state of the cells.

The protein analysis of all the other selected genes showed no differences of expression among genes belonging to the four categories of expression (high, medium, low expression or not expressed). Moreover, cells in which genes had a weak transcript expression produced the protein with a clear and intense signal, such as SMN1 and C9orf72 proteins. To expand the disease spectrum beyond the NMD field, we studied the *HTT* gene, mutations in which cause HD, an autosomal dominant, lethal, neurodegenerative disease (Hong et al., 2021). We showed that the *HTT* gene was well represented both at the transcript and protein level. This finding is very relevant, since USC might be used to model HD, perhaps without any reprogramming (Csobonyeiova et al., 2020).

To conclude, we show for the first time that a variety of NMDs diseases could be successfully studied in USCs. Among 610 NMD genes interrogated by RNA-seq, 93% of them were highly transcribed, and, consistently, all 11 proteins we tested were expressed. The *HTT* gene transcript and protein positivity strongly suggest USCs as a cell tool to study other central nervous system degenerative diseases.

This study shows that USCs may be adopted for basic research and might be also used if validated for diagnostics and clinical applications in the context of rare disease. However, since we focused our study on healthy donors only, further in depth studies are needed to overcome this limitation and to demonstrate that USCs obtained from patients affected with different diseases and carrying specific genes’ mutations may recapitulate the disease-specific cellular phenotype.

We expect that these novel data will provide a resource for developing a new USCs *in vitro* model for several NMDs that will contribute to the understanding of some pathological mechanisms and to the discovery of new therapeutic approaches.

## Data Availability Statement

RNA-seq data for this study have been deposited in the GEO database under accession number GSE162108.

## Ethics Statement

The studies involving human participants were reviewed and approved by the UNIFE Ethical Committee approval, No. 161299 (20/05/2020). The patients/participants provided their written informed consent to participate in this study.

## Author Contributions

MSF and AF designed the study. MSF and RR wrote the manuscript. HO conducted the cell isolation and culturing. MF, ZL, and WL performed RNA-seq raw data analysis. AG, SB, and RR performed bioinformatics and statistical analysis of RNA-seq outputs. ST, PS, and MA performed protein analysis. FG, RS, and CA-K critically revised the manuscript. ST and AF finalized the manuscript. All authors contributed to the article and approved the submitted version.

## Conflict of Interest

AF is the principal investigator of Sarepta Therapeutics Essence and MIS51ON clinical trial for DMD and is the PI of ongoing grants on DMD diagnosis funded by PTC Therapeutics and Sarepta Therapeutics. The remaining authors declare that the research was conducted in the absence of any commercial or financial relationships that could be construed as a potential conflict of interest.

## Publisher’s Note

All claims expressed in this article are solely those of the authors and do not necessarily represent those of their affiliated organizations, or those of the publisher, the editors and the reviewers. Any product that may be evaluated in this article, or claim that may be made by its manufacturer, is not guaranteed or endorsed by the publisher.

## Footnotes

1
http://www.musclegenetable.fr/

2
https://www.clinicaltrials.gov/

3
http://www.bioinformatics.babraham.ac.uk/projects/fastqc/

4
https://www.nature.com/articles/d41586-020-00352-6

## Supplementary Material

Supplementary Table 1 List of identified 20,716 genes expressed in WT-n and WT-m USCs. Genes were categorized into four groups based on FPKM quantiles, i.e., high if the FPKM values were higher than the 90th percentile of the distribution of FPKM values; middle if the FPKM values were comprised between the 80th and the 90th percentile; low if the FPKM values were comprised between the 50th and the 80th percentile; and not expressed if the FPKM values were lower than the median FPKM.

Supplementary Table 2 List of genes causing NMDs (http://www.musclegenetable.fr/, accessed 16 March 2021).

Supplementary Table 3 List of NMD genes expressed in both WT-n and WT-m USCs. The table shows the 571 NMD transcripts expressed in WT USCs with the relative FPKM value, rank and expression category extrapolated from Supplementary Table 1.

Supplementary Table 4 List of 16 categories (A-R) of NMDs. The table shows the FPKM value and the expression category of all genes belonging to the 16 groups of NMDs, comparing WT-n and WT-m USCs.
